# Promoting Mental Health Literacy of 13–16-Year-Old Students: A Systematic Review

**DOI:** 10.3390/ijerph22101578

**Published:** 2025-10-16

**Authors:** Aino Asplund, Maika Kummel, Camilla Laaksonen, Karmen Erjavec, Evanthia Sakellari, Elina Santala, Joonas Korhonen

**Affiliations:** 1 Emergency Care, Public Health Nursing, Midwifery and Diagnostic Services, Faculty of Health and Well-Being, Turku University of Applied Science, 20520 Turku, Finlandjoonas.korhonen@turkuamk.fi (J.K.); 2 Faculty of Health Sciences, University of Novo mesto, 8000 Novo Mesto, Slovenia; karmen.erjavec@uni-nm.si; 3 Laboratory of Hygiene and Epidemiology, Department of Public and Community Health, School of Public Health, University of West Attica, 115-21 Athens, Greece; sakellari@uniwa.gr; 4 Department of Nursing Science, Faculty of Medicine, University of Turku, 20014 Turku, Finland

**Keywords:** mental health literacy, mental health promotion interventions, secondary school students

## Abstract

Purpose: The global rise in mental health challenges among adolescents has led to the development of targeted interventions, including those designed to promote young people’s understanding and awareness of mental health. This systematic review aims to identify and evaluate interventions implemented among secondary school students with the goal of enhancing mental health literacy, and to assess their effectiveness in achieving this outcome. Methods: This review was partially aligned with the PRISMA 2020 guidelines. Studies were retrieved from four electronic databases—CINAHL Complete, PubMed, Academic Search Elite, and ERIC—and supplemented by a limited number of relevant studies identified outside the systematic search. The analysis focused on the content, delivery, design, evaluation methods, and outcomes of mental health literacy (MHL) interventions conducted in secondary schools internationally. Results: A total of 16 articles met the inclusion criteria. The articles described a variety of different MHL interventions (n = 12). The findings indicate that school-based MHL interventions have a positive impact on students’ knowledge and understanding of mental health, but stigma reduction demonstrated less consistent effects. Study population, country, intervention content, delivery, methods, outcome measures, sample sizes and participants’ age range varied notably across the studies. Conclusions: Based on the evidence synthesized in this review, school-based MHL interventions appear to be effective and may serve as a valuable component in broader efforts to promote mental health in adolescence. These findings reinforce existing evidence and provide a foundation for practical implications, but future research is highly recommended for a more solid base of evidence.

## 1. Introduction

Mental health problems pose a significant challenge to the well-being of young people [1,2,3,4,5]. These challenges arise from various reasons, including adverse experiences, peer pressure, and identity-related issues commonly encountered during adolescence [5].

According to a recent meta-analysis, a significant proportion, approximately one-third, of mental health disorder symptoms appear before the age of 14 [6]. Young people’s mental health encompasses their ability to engage in work or education, build and maintain healthy relationships, experience emotions and hope, communicate effectively, and continue learning [5]. It also plays a vital role in shaping self-esteem and overall well-being [7].

If the mental health concerns of young people are not recognized or treated [3], both their mental and physical health may be negatively affected later in life. This may reduce their chances of leading a fulfilling life in adulthood [5].

However, stigma related to mental health issues may prevent adolescents from accessing appropriate support [8,9]. This stigma is often linked to a lack of knowledge regarding mental health conditions [10]. Therefore, early interventions aimed at addressing the factors contributing to mental distress [4] may significantly impact the future well-being of young people [8]. They may also contribute to the sustainability of society, given the burden of mental health conditions on public health [11].

Consequently, it is essential to consider evidence-based, proactive measures to promote mental well-being at early stages. These early interventions may include providing young people with awareness of mental health issues [8] and fostering resilience to help them navigate challenges and life transitions [5].

These elements are central to the concept of mental health literacy (MHL) [12]. A contemporary and evolving understanding of MHL includes knowledge of how to achieve and maintain good mental health, the ability to recognize and understand mental disorders and their treatments, and the capacity to distinguish between typical mood fluctuations and clinical conditions [10,12]. It also encompasses knowing when and where to seek help, as well as cultivating anti-stigma attitudes, including the reduction in both self-stigma and stigma directed toward others [13].

Since its early conceptualization, MHL has incorporated several elements, such as awareness of self-help strategies [14] and coping mechanisms for managing stress [13], which are closely linked to the concept of resilience [15]. Contemporary MHL interventions targeting adolescents often include resilience-building components [16,17], such as problem-solving [16] or the ability to adapt to challenging life events [15,18]. Crucially, adolescents’ resilience is a capacity that can be strengthened over time [15], and higher levels of MHL are associated with greater resilience [19].

Schools are widely acknowledged as key environments for reaching large numbers of students and play a pivotal role in shaping young people’s lives [20]. Adolescence is a critical period for the implementation of these programs, given the emotional [5,21], social, and cognitive [21] transitions that characterize this stage of development [9,16]. Within this context, secondary school-based MHL programs are increasingly recognized as promising approaches to address MHL-related issues, such as reducing stigma and encouraging early help-seeking behaviors [11,22]. Research further suggests that such interventions can significantly enhance students’ understanding of mental health-related issues and foster more accepting attitudes [23,24,25].

Despite a growing body of research supporting the efficacy of MHL programs, several important gaps remain. First, there is a lack of comprehensive reviews that systematically evaluate the impact of MHL interventions specifically among adolescents aged 13–16—a developmentally sensitive age group [9,21]. This age group corresponds to the target population of the project on which this review is based. Second, the literature provides limited insight into the relative effectiveness of different delivery formats, particularly peer-led versus teacher- or professionally led approaches—an area that remains underexplored [26]. Third, although the number of school-based MHL studies has increased, variation in methodological approaches and evaluation tools limits the comparability and generalizability of findings [9,23].

This systematic review aims to

- Identify interventions implemented among secondary school students with the goal of enhancing mental health literacy;
- Evaluate the aforementioned interventions;
- Assess interventions’ effectiveness among secondary school students with the goal of improving mental health literacy.

Focusing on peer-reviewed studies published between 2013 and 2023, it captures recent developments in the field and informs the evidence-based development of contextually appropriate MHL strategies within educational frameworks. The review addresses three core research questions, emphasizing the focus areas and delivery of the MHL interventions; the study design and measurement tools; and the outcomes of the interventions. The analysis is guided by an established conceptual definition of MHL, which includes knowledge of mental health promotion, ability to recognize and understand mental disorders and treatments, supporting help-seeking ability and reducing stigma [10,12,13] while acknowledging that not all included studies explicitly adopt this framework.

## 2. Materials and Methods

### 2.1. Database Sources and Search Strategies

The literature search was conducted following the guidelines of the Preferred Reporting Items for Systematic Reviews and Meta-Analyses (PRISMA) 2020 [27] conducted on 20–21 June 2023. The databases CINAHL Complete, PubMed, Academic Search Elite, and ERIC were used in the search. The following terms were used in the search: “mental health literacy” OR “mental health education” OR “mental health knowledge” AND intervention/“intervention” OR program/“program” OR education/“education” OR method/“method” AND youth/“youth” OR adole/“adole*” OR “young people” OR teen*/“teen*” OR pupil*/“pupil*” AND “secondary school” OR “high school” OR “secondary education” (Table 1). The following search modes were used (not all applicable to all databases): Boolean/Phrase, 1 January 2013–31 December 2023, English language and Text Word. The database search was supplemented with two additional articles via hand search. The literature was reviewed between spring 2024 and spring 2025.

The database search was conducted by one author. Two authors were primarily involved in data extraction after the database search, and these results were further discussed with other authors in line with the inclusion and exclusion criteria to decide whether the article should be included. There were no discrepancies between the authors about data extraction.

### 2.2. Inclusion and Exclusion Criteria

This review focuses on adolescents aged 13 to 16 years, aligning with the target group of the broader European Union co-funded Erasmus+ project *wExchange* (KA220-SCH-A9C42879; https://wexchange.turkuamk.fi/ (accessed on 2 September 2024)), which seeks to promote youth mental health through virtual mobility. The inclusion and exclusion criteria outlined in Table 2 were applied to all identified articles.

### 2.3. Study Selection

**Figure 1:**
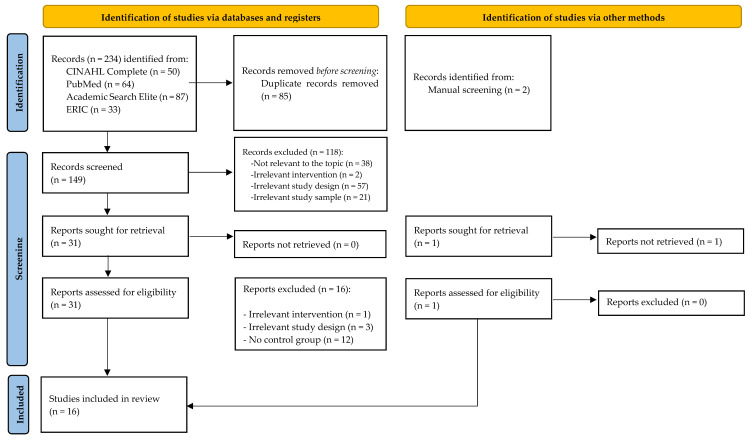
PRISMA flow diagram of the search process (adapted from Page et al. 2021 [27]).

According to Figure 1, 234 articles corresponding to the search terms were initially identified through the selected databases. After the removal of duplicates, a total of 149 articles remained for further screening. The study selection was based on the review of the titles and abstracts of the articles against the eligibility criteria presented in Table 2; 118 were excluded, leaving 31 articles for full-text reading. Of these, 16 articles were excluded due to: irrelevant study design (n = 3), irrelevant intervention (n = 1), and lack of a control group (n = 12). The database search was supplemented with two additional articles via hand search, of which one was excluded due to an irrelevant study design. The other met the inclusion criteria and was added to the number of articles included.

## 3. Results

### 3.1. Characteristics of the Included Articles

A summary of the 16 articles included in the review is presented in Table 3. The articles described 12 different interventions. *Mental Health and High School Curriculum Guide* was described in three of the included studies [11,17,29]. The *Adolescent Depression Awareness Program* (ADAP) was discussed in two studies [30,31]. *It’s Time to Start Talking* (ITTST), *Finding Space for Mental Health*, *teen Mental Health First Aid*, *Innate Health Education and Resilience Training* (iHEART), *Rational Emotive Behaviour Therapy* (REBT), *Guide Cymru*, *Mental Health Matters* (MHM), and the *Short MHL Programme* (SMHLP) [9,32,33,34,35,36,37] each featured in one study. Additionally, three studies described unnamed interventions [8,38,39].

The included articles were published between 2014 and 2023. The studies were conducted in a range of countries across the globe. Correspondingly, interventions were organized in many countries (e.g., in Japan, Egypt, and Nigeria) and thus conducted not only in English but also in other languages. The Mental Health and High School Curriculum Guide intervention was examined in Canada, Iran, and Nicaragua [11,17,29]. All other interventions were studied in a single country each, including Australia [32], Canada [29], Egypt [8], Greece [39], Iran [11], Ireland [26], Japan [37], Nicaragua [17], Nigeria [38], Norway [34], Portugal [9], the UK [33,35], and the USA [30,31,36].

The sample size across the included studies ranged from *N* = 59 [39] to *N* = 6679 [30] and the study population between ages 10 and 25. The participants’ age range was relatively narrow, two to three years, in some of the studies [9,11,30,31,32,37] while some studies included participants across a very broad, eight to eleven, age spectrum [17,38]. 

### 3.2. Focus Areas and Delivery of the Mental Health Literacy Interventions

As outlined in Table 3, the included studies aimed to evaluate the outcomes of several (n = 12) different MHL interventions implemented in secondary school settings.

The interventions *Finding Space for Mental Health* [9], *The Mental Health and High School Curriculum Guide* [11,17,29], the *Short MHL Programme* (SMHLP) [37], *Guide Cymru* [35], *It’s Time to Start Talking* (ITTST) [32], *The Mental Health Matters* [36], the intervention described by Abd El Salam et al. [8] and Bella-Awusah et al. [38] focused on multiple MHL subdomains, including mental health knowledge, stigma reduction, help-seeking, and self-help strategies.

The *Innate Health Education and Resilience Training* (iHEART) program focused on resilience and wellbeing among young people [33], while Sakellari et al. [39] presented an intervention specifically designed to promote adolescents’ positive perceptions of individuals experiencing mental health challenges.

Some of the interventions included did not target general subareas of MHL but focused on specific mental health challenges. The *Adolescent Depression Awareness Program (ADAP)* focused on increasing depression literacy [30,31] and *Rational Emotive Behavior Therapy (REBT)* [34] on increasing self-esteem, hope and reducing anxiety, depression, and dysfunctional behavior. The *Teen Mental Health First Aid* [32] focused on peer support for adolescents at risk of suicide.

The interventions varied in how they were delivered. Several studies (n = 7) described interventions delivered by schoolteachers who had received specific training beforehand [11,17,29,30,31,35,37]. Two studies described peer-led interventions [26,32]. Some interventions were delivered by therapists [34] and psychologists [9], while others by researchers, project staff, trained facilitators [8,33,38,39] or trained community members [36].

The duration of the interventions varied considerably, ranging from a single 40 min workshop [26] to weekly 60 min lessons implemented over 10–12 weeks [17]. *The Mental Health and High School Curriculum Guide* varied in duration across studies [11,17,29]. Most commonly, interventions consisted of two to three lessons or sessions, each lasting between 20 and 90 min [8,9,30,32,34,37,38,39]. The studies highlighted a diverse range of methods, including a website featuring articles and stories [17], lectures [8,11,30], slide shows and panel discussions [8], role-play, self-directed learning [11], animations, exercises, games [33], videos [9,30,33], music [9], question-and-answer activities [11], and collaborative group work [9,11,30,33].

### 3.3. Study Design and Measurement Tools

The study designs were cluster-randomized controlled trials [8,11,32,35,37], randomized controlled trials (RCTs) without a cluster component [9,29,30,31,34] and non-randomized controlled trials [17,26,33,36,38,39].

Most studies (n = 13) adopted a quantitative approach [9,11,17,26,29,30,31,32,34,35,36,37,38], while the remaining (n = 3) employed a mixed-methods approach [8,33,39].

A variety of quantitative and qualitative measurement tools were employed to assess the outcomes of the respective interventions. In addition to demographic questionnaires and study-specific, author-developed tools [11], several (n = 13) previously developed and validated questionnaires were identified across the studies [8,9,17,26,30,31,32,33,34,35,36,38].

As summary, most studies employed different measurement tools to assess similar outcomes. Only a few instruments, the Mental Health Literacy Questionnaire (MHLq) [9,32], the Adolescent Depression Knowledge Questionnaire, and the Reported and Intended Behavior Scale [30,31] were used in more than one study. Only a few studies reported follow-up testing (n = 5). Time of follow-up varied between three [17], four [30] and six [8,9,34] months.

### 3.4. Outcomes of the Interventions

According to this systematic review, MHL interventions achieved several positive outcomes; however, some results were modest or limited in their effectiveness (Table 3). The reported interventions increased secondary school students’ knowledge of mental health [9,11,17,26,29,35,36,37,38], help-seeking and [8,9,26,35,37] self-help abilities [9,11], reduced stigma [8,11,35,36,39], and supported impulse control and resilience [33].

Studies focusing on interventions targeting specific mental health challenges also reported positive outcomes. Students reported being more likely to recognize suicidality in a peer and provide appropriate help [32], reported reduced symptoms of depression and anxiety, and increased self-esteem and hope [34], as well as improved depression literacy [30]. Some studies reported no or modest effects on attitudes, social distance [38], and stigma [17,30,35].

When examining specific aspects of MHL more closely, six studies [8,9,11,17,30,35] addressed issues related to mental health knowledge, with Swartz et al. [30] focusing on depression literacy. All the referenced studies indicated positive findings in increasing mental health knowledge, covering information on mental health, causes of mental illness, and specific mental health conditions [11] as well as their symptoms [30]. Additionally, Abd El Salam et al. [8] reported positive results in altering potentially culture-related perceptions of mental health issues as being due to supernatural powers, with students showing a significant decrease in their belief that mental illness is caused by evil spirits (*p* < 0.001).

The importance of different interventions in contributing to help-seeking preferences was also evident [8,9,11,35]. Despite positive findings regarding the increase in help-seeking, Abd El Salam et al. [8] found an adverse outcome when students sought help from their peers rather than professionals, thereby risking the spread of misleading information about mental health. However, Ravindran et al. [17] did not find a significant difference between the intervention and control groups in help-seeking tendencies. Campos et al. [9] suggested that girls are more willing to seek help for mental health conditions than boys, even though gender did not significantly affect the intervention’s effectiveness.

Regarding resilience, the effectiveness of the interventions remains inconclusive. The Mental Health Curriculum study by Ravindran et al. [17] found no significant differences between the intervention and control groups in terms of improving resilience. Similarly, the study by Kelley et al. [33] indicated that the “iHEART” intervention did not lead to significant improvements in resilience when assessed using quantitative methods (r = 0.14). However, qualitative data yielded more promising results.

The interventions varied in their effectiveness in addressing mental health-related stigma. Several studies [8,9,11,17,35] reported positive effects of MHL interventions in reducing stigma. Swartz et al. [30] did not report any significant impact on stigma while Simkiss et al. [35] observed improvements in stigma towards others (*p* < 0.001) but found no clear evidence on effectiveness in addressing self-stigma (*p* = 0.59). Nevertheless, the study highlighted the importance of MHL interventions in increasing young people’s willingness to talk about mental health. Abd El Salam et al. [8] also found that, despite overall positive changes in attitudes through education, students remained unwilling to marry a person with a mental illness. Campos et al. [9] reported greater intervention effectiveness in reducing stereotypes among individuals who knew someone with a mental illness.

## 4. Discussion

### 4.1. Summary of Findings and Comparison with Previous Studies

This systematic review aimed to identify and evaluate interventions implemented among secondary school students with the goal of enhancing mental health literacy, and to assess their effectiveness.

In summary, it can be concluded that a total of 16 articles met the inclusion criteria. The articles described a variety of different MHL interventions (n = 12) and took place in various countries worldwide. Several interventions focused on multiple MHL subdomains, but some of them focused on specific mental health challenges. The interventions differed both in how they were delivered and, in their duration, which varied considerably. The studies included cluster-randomized controlled trials, standard randomized controlled trials, and non-randomized controlled trials. A variety of quantitative and qualitative measurement tools were employed to assess the outcomes of the respective interventions. School-based MHL interventions generally increased secondary school students’ knowledge of mental health [9,11,17,26,29,35,36,37,38], help-seeking and self-help abilities [8,9,11,26,35,37], reduced stigma [8,11,35,36,39], and supported impulse control and resilience [33]. These findings are in line with previous research [11,22,23,24,25].

Improved mental health knowledge enhanced understanding of mental health issues [9,11,17,35], including more specific acceptance of mental health concerns within oneself and others [35]. Interventions also helped conceptualize mental illness similarly to other medical conditions and challenged non-evidence-based assumptions about mental health [8].

Not all outcomes were consistently positive, particularly in relation to stigma. Some interventions showed positive results in reducing stigma [8,17,29,35] whereas some showed no effect [30,36]. This finding is consistent with a broader societal reluctance to discuss mental health issues openly, despite efforts by professionals to frame mental illnesses as equivalent to physical health conditions [7]. The mixed results found in this review reflect previous studies reporting both effective stigma reduction [24,25] and inconsistent outcomes [40]. Self-stigma was identified as more resistant to change than stigma towards others [35].

Stigma is a major barrier for help-seeking [13,41] and stigmatizing attitudes were found to stem from various factors, including religious beliefs, supernatural interpretations, and limited access to evidence-based information [13]. Increased mental health knowledge and familiarity with mental health conditions play key roles in stigma reduction [13,41].

Additional cultural and societal barriers—such as concerns about confidentiality or a lack of exposure to mental health discussions—can further discourage help-seeking [9,40]. In some cases, the presence of mental health problems within families was linked to increased secrecy and reluctance to talk openly [42].

It can be concluded, that the results demonstrate considerable heterogeneity. Some outcomes, such as knowledge and general stigma reduction, were consistently positive, while others, such as attitudes and self-stigma, showed mixed or limited effects. Differences also arose based on the type of intervention, who delivered it, cultural and gender factors, and whether the intervention targeted general mental health literacy or specific challenges.

Comparing effectiveness between the identified, school-based MHL interventions is difficult as notable variation emerged in focus areas, specific aims and implementation of the interventions as well as study designs, settings, population, participants and outcome measures. Most studies used previously validated measurement tools, but surprisingly, some of these tools were used in different studies to assess different outcomes. Moreover, some articles lacked a clear description of the research methodology resulting in the impossibility of assessing the quality of the data and validity of the reported results.

### 4.2. Implication for Practice and Future Research Directions

The studies reviewed were conducted in a variety of sociocultural contexts (e.g., Iran, the UK, Nicaragua, Egypt, the USA, Portugal), underscoring how cultural norms, values, and health beliefs shape the implementation and reception of MHL interventions. One may speculate that deeply rooted cultural factors associated with stigma may necessitate more context-specific anti-stigma strategies whereas promoting knowledge and help-seeking behaviors may be more universally transferable. Future research would benefit from directly comparing culturally adapted and standardized MHL interventions, along with their culturally validated measurement tools, across different regions. Additionally, it would be valuable to examine which components, such as peer-led sessions, teacher facilitation, or community involvement, are most effective in specific cultural contexts. It would also be recommended to assess teacher- and professional pathways as well as gender-specific effects of MHL interventions in different socio-cultural environments.

While randomized controlled trials (RCTs) are recommended for evaluating intervention effectiveness [43,44,45], the exclusion of studies without control groups may reduce the diversity of evidence and introduce a bias favoring RCTs and non-randomized controlled trials (NRCTs), potentially overlooking valuable insights from alternative study designs. Consequently, some potentially relevant pre–post studies were not included in the review, which may have impacted on the comprehensiveness of the findings. Nevertheless, to determine long-term impacts and the sustainability of the outcomes, further follow-up studies are required. Many interventions demonstrated short-term benefits, but only a few studies assessed outcomes beyond several months. Longitudinal well established RCTs including follow-ups, ideally over multiple years, are essential to capture sustained changes in knowledge, behavior, stigma, resilience, and other psychosocial outcomes evolving throughout adolescence.

Peer-led approaches have shown promise in enhancing MHL [23,40]. However, only a few peer-led interventions were identified in this review, highlighting a notable gap for further exploration. Future research should compare the outcomes of peer-led interventions with those led by teachers or professional facilitators to determine their relative effectiveness and feasibility. Such comparisons would help clarify whether peer-led models can improve engagement, reduce stigma, and contribute meaningfully to sustainable MHL initiatives in secondary schools.

Although school-based MHL interventions are often seen as cost-effective due to their broad reach and use of existing infrastructure, their long-term viability warrants closer examination. Teachers may require specialized training and support, and updating program materials can add costs. Sustaining these initiatives beyond the research phase is also challenging. Without continued funding or institutional backing, benefits may fade.

### 4.3. Strengths and Limitations

One of the strengths of this review lies in a relatively high number of included studies (n = 16) and the contribution to the previous evidence base concerning varied impacts of MHL interventions. Notably, the literature search applied no geographical restrictions, thereby incorporating evidence from a wide range of countries across different continents. In addition, the inclusion of diverse study designs may offer a broader insight into the effectiveness of MHL interventions.

While this review provides valuable insights into school-based MHL interventions, several limitations must be acknowledged. The wide variety of interventions, methodologies and measurement tools used to assess similar concepts, causes evidence to be scattered. The heterogeneity in definitions of MHL and the varying components of interventions across studies may impede robust meta-level conclusions. Establishing consensus on core MHL constructs would support more accurate comparisons of outcomes. Moreover, the validity of exclusion of studies, lacking a control group design, may have led to the omission of potentially strong evidence, thus narrowing the analytical scope of this review. It may be considered that this review was affected by publication and cultural bias. Because studies with strong research methods were selected and differences in available resources exist between countries, not all regions or backgrounds may be represented. As a result, the perspectives and contexts represented may be limited.

One may also point out the wide variety of study participant number and age range as potential limitations on validity of the summarized study outcomes. The review was limited to studies published primarily in English, potentially excluding effective interventions reported in other languages or in the gray literature.

Future systematic reviews should aim to expand their scope by searching additional databases, extend the publication timeframe, and explicitly focus on specific features, such as peer-led MHL programs to address current gaps in the literature.

## 5. Conclusions

Based on the evidence synthesized in this review, school-based mental health literacy (MHL) interventions appear to be effective and may serve as a valuable component in broader efforts to promote adolescent mental health. These findings reinforce existing evidence and provide a foundation for practical implications.

This review highlights various factors, including lesson plan standardization, peer-support elements, and curricular integration. These factors can inform the practical design and implementation of MHL programs in secondary schools. The evidence is, however, still scattered. Several school-based MHL interventions have been identified, but the effectiveness of these interventions has been reported mainly in only one or very few studies. Future research should adopt longitudinal follow-ups and solid research designs and methods to validate and extend findings, ensuring that MHL initiatives remain both feasible and effective in diverse educational settings.

## Figures and Tables

**Table 1:** Search terms used in different databases.

Database	Search Terms Used
CINAHL Complete	“mental health literacy” OR “mental health education” OR “mental health knowledge” AND intervention/”intervention” OR program/”program” OR education/”education” OR method/”method” AND youth/”youth” OR adole/”adole” OR “young people” OR teen/”teen” OR pupil/”pupil*” AND “secondary school” OR “high school” OR “secondary education”
PubMed	“mental health literacy” OR “mental health education” OR “mental health knowledge” AND intervention/”intervention” OR program/”program” OR education/”education” OR method/”method” AND youth/”youth” OR adole/”adole” OR “young people” OR teen/”teen” OR pupil/”pupil*” AND “secondary school” OR “high school” OR “secondary education”
Academic Search Elite	“mental health literacy” OR “mental health education” OR “mental health knowledge” AND intervention/”intervention” OR program/”program” OR education/”education” OR method/”method” AND youth/”youth” OR adole/”adole” OR “young people” OR teen/”teen” OR pupil/”pupil*” AND “secondary school” OR “high school” OR “secondary education”
ERIC	“mental health literacy” OR “mental health education” OR “mental health knowledge” AND intervention/”intervention” OR program/”program” OR education/”education” OR method/”method” AND youth/”youth” OR adole/”adole” OR “young people” OR teen/”teen” OR pupil/”pupil*” AND “secondary school” OR “high school” OR “secondary education”

**Table 2:** Inclusion and Exclusion Criteria (adapted from Gierisch et al. [28]).

Study Characteristic	Inclusion Criteria	Exclusion Criteria
Study design	Intervention studies	Non-experimental studies, editorials and/or commentaries, book reviews and/or letters, dissertations, study protocols
Population	Includes secondary school students aged 13 to 16 years	Includes other than secondary school students between age 13 to 16
Intervention	Mental health literacy promotion	General mental health promotion, diagnostics, care or rehabilitation of mental health challenges/illnesses, therapeutic interventions
Comparators	Any other ordinary intervention/no intervention	No control group
Setting	Secondary school	Any other setting
Outcomes	Mental health literacy, sub-areas of mental-health literacy	Any other outcomes
Other	Articles published between 2013 and 2023, peer-reviewed, published in English language	Articles published before 2013 or after 2023, not peer-reviewed, published in languages other than English

**Table 3:** Review of the final studies.

Author/Year/Country	Study Focus	Study Design and Population	Name, focus and delivery of Intervention	Duration of Intervention	Evaluation Tools	Intervention Outcomes
Abd El Salam et al. (2023) Egypt	Evaluate impact of the intervention on students’ knowledge and attitudes towards mental illness and perceptions on help-seeking.	CRCT Pre-test: n = 416 Post-test: n = 224 (allocated to intervention and control groups) Age of the participants (years): 13–18	Unnamed intervention Focus: Stigma Mental illness Barriers to help-seeking Methods: A didactic component, group discussions, homework exercises, videos, lectures, slide shows and panel discussions. Delivery: Teacher led	3 lessons/week under 1 month 20–30 min/lesson	Adapted version of MINI international neuropsychiatric interview for children and adolescent questionnaire Pre–post test (6 month follow up)	Positive effects on the beliefs of mental illness being like any other illness, the incorrect belief of mental illness being an evil spirit. Decreased stigma (except for marrying a person with mental illness) Positive effects on willingness to seek help from family members or healthcare workers
Bella-Awusah et al. (2014) Nigeria	Evaluate impact of the intervention on mental health literacy and reducing negative views about persons with mental illness	NRCT Intervention group n = 78 Control group n = 76 Age of the participants (years): 10–18	Unnamed intervention Focus: Views on mental health Behaviors indicating mh challenges Understand limitations regarding responsibility Ways to support peers Strategies to promote mh Methods: Group work, discussions, presentations, case vignettes Delivery: two project staff	A 3 h mental health awareness session.	Modified version of the UK Pinfold questionnaire. Statements were added to the original questionnaire based on myths and beliefs surrounding mental illness in the Nigerian context.	Small positive changes in knowledge but not in attitudes and social distance.
Booth et al. (2023) Ireland	Evaluate impact of the intervention on mental health literacy and reducing negative views about persons with mental illness.	NRCT Peer-led workshops n = 245 Adult-led workshops n = 291 Age of the participants (years): 12–17	It’s Time to Start Talking (ITTST) Focus: Attitudes to mental health, Promote help-seeking Identify trusted informal sources of support Information about how to access formal support Methods: Workshop Delivery: Peer-led, adult-led	40 min workshop	Author-designed questionnaire The General Help Seeking Questionnaire	Mental health knowledge and help-seeking intentions improved in both peer- and adult-led groups
Campos et al. (2018) Portugal	Evaluate impact of the intervention on mental health literacy	RCT Intervention group n = 259 Control group n = 284 Age of the participants (years): 12–14	Finding Space for Mental Health Focus: Mental health knowledge Stigma Help-seeking First-aid skills Self-help Methods: Group dynamics, music, videos Delivery: Psychologist and a psychology student	2 lessons (2 weeks) 90 min/lesson	Mental Health Literacy questionnaire (MHLq) Pre–post test (1 week, 6 months after intervention)	Intervention group showed higher improvement in MHL when compared to the control group Gender differences: Boys showed less intention to seek help and to help someone in need compared to girls
Hart et al. (2020) Australia	Evaluate impact of the intervention on peer support for adolescents at risk of suicide and examine whether participation in a program is distressing to participants.	CRCT Control group (physical first aid) n = 790 Intervention group: n = 807 Age of the participants (years): 15–17	Teen Mental Health First Aid aimed Focus: Peer support towards adolescents at risk of suicide Methods: presentation, training, videos, role-plays, group discussion, small group and workbook activities Delivery: Trained extractor	3 × 75 min classroom sessions (within 3 weeks)	A questionnaire including items adapted from the Australian National Survey of Youth Mental Health Literacy	Positive outcomes on recognition of suicidality and appropriate first aid intentions towards a peer at risk of suicide 12 months after training, most effects were still significant. Intervention participants reported feeling briefly distressed after the training, but not at 12 months follow up
Kelley et al. (2021) UK	Evaluate impact of the intervention on mental well-being and resilience.	NRCT Intervention group n = 205 Control group n = 64 Age of the participants (years): 11–15	Innate Health Education and Resilience training (iHEART) Focus: Information on resilience and mental wellbeing Methods: Animations, video clips, exercises, games, group activities Delivery: trained facilitator	10 consecutive weeks 50 min/lesson	Warwick-Edinburgh Mental Well-being Scale (WEMWBS) Inside-Out Resilience Questionnaire (I-ORQ) Pre–post test 3 questions on participants’ perceptions of the intervention: resilience and mental well-being Post test only	Positive effects on mental well-being, resilience and impulse control
Milin et al. (2016) Canada	Evaluate impact of the intervention on mental health knowledge and stigma.	RCT Intervention group n = 362 Control group n = 172 Age of the participants (years): mean age 16.5	The Mental Health and High School Curriculum Focus: Understanding of mental health and illness Reduce stigma Promote help-seeking behaviors Methods: Curriculum Guide of 6 modules. These modules contain a lesson plan embedded classroom activities and resources	~6 h (mainly within 4 weeks)	Questionnaires on primary outcome measures of mental health knowledge, attitudes and stigma Pre–post test	Positive effects on mental health knowledge and reduction in stigma
Miller et al. (2019) USA	Evaluate impact of the intervention on depression literacy and stigma of teachers and their students.	RCT Teachers n = 66 Students n = 6679 Intervention group n = not reported Control group n = not reported Age of the participating students (years): 14–16	Adolescent Depression Awareness Program (ADAP) Focus: Support treatment-seeking behavior Reduce stigma Increasing depression literacy Methods: depression education as part of the standard high school health education curriculum Delivery: Teacher led	Teachers received 6 h ADAP-manualized training: included program and medical overview, implementation instructions, education kit (teaching manual, PP-lectures, group activities, handouts and DVDs.) ADAP intervention was administered to students as part of 2–3 of the standard health curriculum classes.	The Adolescent Depression Knowledge Questionnaire (ADKQ) The Reported and Intended Behavior Scale (RIBS) Pre–post test	Teacher depression literacy was associated with student depression literacy but not with student stigma. Teacher stigma was not related to student depression literacy or stigma
Ravindran et al. (2018) Nicaragua	Evaluate impact of intervention on knowledge, stigma, psychological distress, substance use, stress, resilience and quality of life.	NRCT Intervention group n = 567 Control group n = 346 Age of the participants (years): 14–25	Mental Health Curriculum (MHC) Focus: Stigma Knowledge on mental health and illness Experiences of mental illness Help-seeking Positive mental health Methods: Education for teachers and students, guide for teachers, website on MHC (articles, presentations, videos, stories), message board forums Delivery: Teacher led	12 consecutive weeks 60 min/lesson	Mental Health Knowledge and Attitudes Scale (MHKAS) Attitudes subscale The Brief COPE (coping strategies) The CRAFT (alcohol and drugs) The General Health Questionnaire—12 (GHQ-12) The Health-Promoting Lifestyle Profile II (HPLP II) The Perceived Stress Scale (PSS) The Quality of Life Scale (QOL) The Resilience Scale—Short Form (RS-14) Pre–post test (12 weeks)	Positive effects on mental health knowledge, healthy lifestyle choices, adaptive coping, reduction in stigma and perceived stress
Sakellari et al. (2014) Greece	Evaluate impact of intervention on perceptions of people with mental illness through drawings, describe these perceptions, and test the possible changes in perceptions after an educational mental health intervention	NRCT Intervention group n = 28 Control group n = 31 Age of the participants (years): 13–16	Unnamed intervention Focus: Knowledge and understanding of mental health and illness Prevention and treatment of mental illness Myths and truths about mental health Methods: Education, discussion, messages to take home. Delivery: Teacher led	2 teaching hours (approximately 60 min of presentation and 30 min of discussion)	Drawings on people with mental illness Pre–post test	Positive effects on presenting people (in the drawings) with mental illness
Simkiss et al. (2023) UK	Evaluate impact of intervention on mental health knowledge, stigma and help-seeking	CRCT Total N = 1926 Age of the participants (years): 13–14	The Guide Cymru Focus: Knowledge of mental health and illness Stigma Experiences of mental illness Help-seeking and finding support Positive mental health Methods: Not described in the article Delivery: teacher led	10–12 weeks	The Knowledge and Attitudes to Mental Health Scales (KAMHS) Pre–post test	Positive effects on mental health knowledge and behavior, intentions to seek help and avoidant coping Positive effects on stigma to others and self-stigma even though result on self-stigma was less clear
Sælid and Nordahl (2017) Norway	Evaluate and compare impacts of interventions on self-esteem and hope, reducing symptoms of anxiety, depression, and dysfunctional thinking	RCT REBT group n = 21 ATP group n = 21 Control group n = 20. Age of the participants (years): 16–19	Rational emotive behavior therapy REBT Focus: Recognize distressing thinking, behavior and beliefs Methods: Therapeutical sessions Delivery: Therapist	REBT: 3 sessions, ATP: 3 sessions	Hospital Anxiety and Depression Scale (HADS) The Rosenberg Self-Esteem Scale (RSES) Herth Hope Index (HHI) Dysfunctional Attitude Scale (DAS-A) Satisfaction evaluation. Pre–post test (6 month)	Both interventions reduced symptoms of anxiety and depression, increased self-esteem and hope. Only REBT reduced dysfunctional thinking
Swartz et al. (2017) USA	Evaluate effects of intervention on depression literacy, sustainability of change and receipt of mental health treatment	RCT Intervention group n = 3681 Control group n = 2998 Age of the participants (years): 14–15	Adolescent Depression Awareness Program (ADAP) Focus: Information about depression symptoms and treatment Understanding depression as a medical illness similar to other illnesses Understanding connection between depression and suicide Decreasing stigma Methods: interactive lectures as part of health education, videos, film assignments, homework, group activities and teaching kit (e.g., instructor’s manual) Delivery: Teacher led	2–3 lessons (3 h in total) 45–90 min/lesson	Adolescent Depression Knowledge Questionnaire (ADKQ) Reported and Intended Behavior Scale (RIBS) Pre–post test (6 week, 4 month) Child and Adolescent Services Assessment 4-month post test only	Positive effects on depression literacy. No effects on stigma
Weisman et al. (2016) USA	Evaluate effects of intervention on mental health knowledge and stigma	NRCT Study 1. Teachers n = 7 Students, intervention group n = 142 Age of the students (years): 11–13 Study 2. Students n =120 Intervention group n = unclear Control group n = unclear Age of the students (years): 12–14	The Mental Health Matters (MHM) Focus: Knowledge of mental health and illness Decrease stigma Methods: curricula included interactive language arts activities, theater play, handouts, home- and groupwork, planning a poster on a mental health disorder, note-taking and a game. Delivery: community volunteers	5-day sessions	Knowledge (19- item test) Revised Attribution Questionnaire (rAQ), Attitudes Toward Serious Mental Illness Scale Adolescent Version ATSMI-AV Open ended questions Pre–post test Evaluation form on acceptability of the intervention Post test only	MHM was perceived acceptable Positive effects on knowledge. Controversial results on reduction in stigma
Yamaguchi et al. (2020) Japan	Evaluate effects of the intervention on knowledge of mental health and illness, help-seeking and peer support	CRCT Intervention group n = 364 Control group n = 611 Age of the participants (years): 15–16	Short MHL Program Focus: Knowledge on common mental disorders and symptoms Mental health problems closely associated with lifestyle Seeking help from reliable adults Peer-support skills Methods: 2 animated films, class discussions Delivery: a health care teacher and class teacher	Two 50 min sessions	Questions regarding general knowledge about mental health and illnesses 2 vignettes describing cases regarding depression and schizophrenia. The vignettes were adapted from those in Jorm et al. (1997). After reading the 2 vignettes, students were asked questions related to the topics. Pre–post test	Positive effects on knowledge, recognition of the necessity of seeking help, intentions of seeking help and helping peers
Zare et al. (2021) Iran	Evaluate effects of the intervention on mental health promotive behaviors	RCT Intervention group n = 110 Control group n = 110 Age of the participants (years): 13–15	Mental Health and High School Curriculum Guide Focus: Information on mental health and illness Stigma Help-seeking/support Positive mental health Self-help Methods: lectures based on interaction, group activities, role-play, independent learning Delivery: Teacher led	6 lessons (6 weeks) 60–90 min/lesson	MHL questionnaire	Positive effects on overall MHL, knowledge, reducing stereotypes and self-help strategies

Abbreviations: MHL mental health literacy, RCT Randomized Controlled Trial, CRCT Cluster Randomized Controlled Trial, NRCT Non-randomized controlled trial.

## References

[1] GBD 2019 Mental Disorders Collaborators (2022). Global, regional, and national burden of 12 mental disorders in 204 countries and territories, 1990–2019: A systematic analysis for the Global Burden of Disease Study 2019. Lancet Psychiatry.

[2] Erskine H.E., Moffitt T.E., Copeland W.E., Costello E.J., Ferrari A.J., Patton G., Degenhardt L., Vos T., Whiteford H.A., Scott J.G. (2015). A heavy burden on young minds: The global burden of mental and substance use disorders in children and youth. Psychol. Med..

[3] Simkiss N.J., Gray N.S., Malone G., Kemp A., Snowden R.J. (2020). Improving mental health literacy in year 9 high school children across Wales: A protocol for a randomised control treatment trial (RCT) of a mental health literacy programme across an entire country. BMC Public Health.

[4] World Health Organisation (2021). Comprehensive Mental Health Action Plan 2013–2030. https://iris.who.int/server/api/core/bitstreams/69921758-6229-49ba-bd3d-c24736e35829/content.

[5] World Health Organization (2024). Mental Health of Adolescents. https://www.who.int/news-room/fact-sheets/detail/adolescent-mental-health.

[6] Solmi M., Radua J., Olivola M., Croce E., Soardo L., Salazar de Pablo G., Il Shin J., Kirkbride J.B., Jones P., Kim J.H. (2022). Age at onset of mental disorders worldwide: Large-scale meta-analysis of 192 epidemiological studies. Mol. Psychiatry.

[7] Njoku I. What Is Mental Illness?. 2025..

[8] Abd El Salam A.E., AbdAllah A.M., El Maghawry H.A. (2023). Effect of health education program on improving knowledge and atitude towards mental health stigma and professional help-seeking among adolescents. Middle East Curr. Psychiatry.

[9] Campos L., Dias P., Duarte A., Veiga E., Dias C.C., Palha F. (2018). Is it possible to “Find space for mental health” in young people? Effectiveness of a school-based mental health literacy promotion program. Int. J. Environ. Res. Public Health.

[10] Kutcher S., Wei Y., Coniglio C. (2016). Mental Health Literacy: Past, Present, and Future. Can. J. Psychiatry.

[11] Zare S., Kaveh M.H., Ghanizadeh A., Asadollahi A., Nazari M. (2021). Promoting mental health literacy in female students: A school-based educational intervention. Health Educ. J..

[12] Jorm A.F. (2019). The concept of mental health literacy. International Handbook of Health Literacy: Research, Practice and Policy Across the Life-Span.

[13] Kutcher S., Wei Y. (2017). Mental Health & High School Curriculum Guide. Understanding Mental Health and Mental Illness.

[14] Jorm A.F. (2000). Mental health literacy. Public knowledge and beliefs about mental disorders. Br. J. Psychiatry.

[15] (2024). American Psychological Association: Resilience. https://www.apa.org/topics/resilience.

[16] Martínez-García A. (2022). Contributions of universal school-based mental health promotion to the wellbeing of adolescents and preadolescents: A systematic review of educational interventions. Health Educ..

[17] Ravindran A.V., Herrera A., da Silva T.L., Henderson J., Castrillo M.E., Kutcher S. (2018). Evaluating the benefits of a youth mental health curriculum for students in Nicaragua: A parallel-group, controlled pilot investigation. Glob. Ment. Health.

[18] Song L., Wang Y., Zhang Q., Yin J., Gan W., Shang S., Qi L., Chen S., Liu T. (2023). The mediating effect of resilience on mental health literacy and positive coping style among Chinese empty nesters: A cross-sectional study. Front. Psychol..

[19] Zhang X., Yue H., Hao X., Liu X., Bao H. (2023). Exploring the relationship between mental health literacy and psychological distress in adolescents: A moderated mediation model. Prev. Med. Rep..

[20] World Health Organization (2017). Health Promoting Schools: An Effective Approach to Early Action on Noncommunicable Disease Risk Factors. https://iris.who.int/server/api/core/bitstreams/01cc4cc4-42b8-4332-b416-ee8995720605/content.

[21] Rampazzo L., Mirandola R., Davis R., Carbone S., Mocanu A., Campion J., Carta M.G., Daníelsdóttir S., Matloňová Z., Holte A. (2016). Joint Action on Mental Health and Well-Being: Mental Health and Schools–Situation Analysis and Recommendations for Action.

[22] Bale J., Grové C., Costello S. (2020). Building a mental health literacy model and verbal scale for children: Results of a Delphi study. Child. Youth Serv. Rev..

[23] Hart L.M., Mason R.J., Kelly C.M., Cvetkovski S., Jorm A.F. (2016). “teen Mental Health First Aid”: A description of the program and an initial evaluation. Int. J. Ment. Health Syst..

[24] Kutcher S., Wei Y., Morgan C. (2015). Successful Application of a Canadian Mental Health Curriculum Resource by Usual Classroom Teachers in Significantly and Sustainably Improving Student Mental Health Literacy. Can. J. Psychiatry.

[25] Lanfredi M., Macis A., Ferrari C., Rillosi L., Ughi E.C., Fanetti A., Younis N., Cadei L., Gallizioli C., Uggeri G. (2019). Effects of education and social contact on mental health-related stigma among high-school students. Psychiatry Res..

[26] Booth A., Elizabeth D., O’Reilly A. (2023). School-based health promotion to improve mental health literacy: A comparative study of peer- versus adult-led delivery. J. Ment. Health.

[27] Page M.J., McKenzie J.E., Bossuyt P.M., Boutron I., Hoffmann T.C., Mulrow C.D., Shamseer L., Tetzlaff J.M., Akl E.A., Brennan S.E. (2021). The PRISMA 2020 statement: An updated guideline for reporting systematic reviews. Syst. Rev..

[28] Gierisch J.M., Bastian L.A., Calhoun P.S., McDuffie J.R., Williams J.W. (2010). Comparative Effectiveness of Smoking Cessation Treatments for Patients with Depression: A Systematic Review and Meta-Analysis of the Evidence.

[29] Milin R., Kutcher S., Lewis S.P., Walker S., Wei Y., Ferrill N., Armstrong M.A. (2016). Impact of a Mental Health Curriculum on Knowledge and Stigma Among High School Students: A Randomized Controlled Trial. J. Am. Acad. Child Adolesc. Psychiatry.

[30] Swartz K., Musci R.J., Beaudry M.B., Heley K., Miller L., Alfes C., Townsend L., Thornicroft G., Wilcox H.C. (2017). School-based curriculum to improve depression literacy among US secondary school students: A randomized effectiveness trial. Am. J. Public Health.

[31] Miller L., Musci R., D’Agati D., Alfes C., Beaudry M.B., Swartz K., Wilcox H. (2019). Teacher Mental Health Literacy is Associated with Student Literacy in the Adolescent Depression Awareness Program. Sch. Ment. Health.

[32] Hart L.M., Cropper P., Morgan A.J., Kelly C.M., Jorm A.F. (2020). Teen Mental Health First Aid as a school-based intervention for improving peer support of adolescents at risk of suicide: Outcomes from a cluster randomised crossover trial. Aust. New Zealand J. Psychiatry.

[33] Kelley T., Kessel A., Collings R., Rubenstein B., Monnickendam C., Solomon A. (2021). Evaluation of the iHEART mental health education programme on resilience and well-being of UK secondary school adolescents. J. Public Ment. Health.

[34] Sælid G.A., Nordahl H.M. (2017). Rational emotive behaviour therapy in high schools to educate in mental health and empower youth health. A randomized controlled study of a brief intervention. Cogn. Behav. Ther..

[35] Simkiss N.J., Gray N.S., Kemp A.H., Dunne C., Snowden R.J. (2023). A randomised controlled trial evaluating the Guide Cymru mental health literacy intervention programme in year 9 (age 13–14) school pupils in Wales. BMC Public Health.

[36] Weisman H.L., Kia-Keating M., Lippincott A., Taylor Z., Zheng J. (2016). Mental Health Stigma Prevention: Pilot Testing a Novel, Language Arts Curriculum-Based Approach for Youth. J. Sch. Health.

[37] Yamaguchi S., Ojio Y., Foo J.C., Michigami E., Usami S., Fuyama T., Onuma K., Oshima N., Ando S., Togo F. (2020). A quasi-cluster randomized controlled trial of a classroom-based mental health literacy educational intervention to promote knowledge and help-seeking/helping behavior in adolescents. J. Adolesc..

[38] Bella-Awusah T., Adedokun B., Dogra N., Omigbodun O. (2014). The impact of a mental health teaching programme on rural and urban secondary school students’ perceptions of mental illness in southwest Nigeria. J. Child Adolesc. Ment. Health.

[39] Sakellari E., Lehtonen K., Sourander A., Kalokerinou-Anagnostopoulou A., Leino-Kilpi H. (2014). Greek adolescents’ views of people with mental illness through drawings: Mental health education’s impact. Nurs. Health Sci..

[40] Hart L.M., Bond K.S., Morgan A.J., Rossetto A., Cottrill F.A., Kelly C.M., Jorm A.F. (2019). Teen Mental Health First Aid for years 7-9: A description of the program and an initial evaluation. Int. J. Ment. Health Syst..

[41] Gulliver A., Griffiths K.M., Christensen H. (2010). Perceived barriers and facilitators to mental health help-seeking in young people: A systematic review. BMC Psychiatry.

[42] Grove C., Reupert A., Maybery D. (2015). Gaining knowledge about parental mental illness: How does it empower children?. Child Fam. Soc. Work.

[43] Gerstein H.C., McMurray J., Holman R.R. (2019). The importance of randomised vs. non-randomised trials–Authors’ reply. Lancet.

[44] Hariton E., Locascio J.J. (2018). Randomised controlled trials–the gold standard for effectiveness research. Int. J. Obstet. Gynaecology.

[45] Hausner E., Metzendorf M.-I., Richter B., Lotz F., Waffenschmidt S. (2018). Study filters for non-randomized studies of interventions consistently lacked sensitivity upon external validation. BMC Med. Res. Methodol..

[46] (2017). American Psychological Association: Ethical Principles of Psychologists and Code of Conduct. https://www.apa.org/ethics/code.

[47] World Medical Association (2013). World Medical Association Declaration of Helsinki. J. Am. Med. Association.

[48] (2021). International Council of Nurses: The ICN Code of Ethics for Nurses. International Council of Nurses. https://www.icn.ch/sites/default/files/2023-06/ICN_Code-of-Ethics_EN_Web.pdf.

